# Supplementary material for: Nigritoxin is a bacterial toxin for crustaceans and insects
Source: Nat Commun. 2017 Nov 1;8:1248. doi: 10.1038/s41467-017-01445-z (PMC5665878; doi:10.1038/s41467-017-01445-z)
Supplement: Supplementary file 1 — Supplementary Information [file 41467_2017_1445_MOESM1_ESM.pdf]

Supplementary Figures

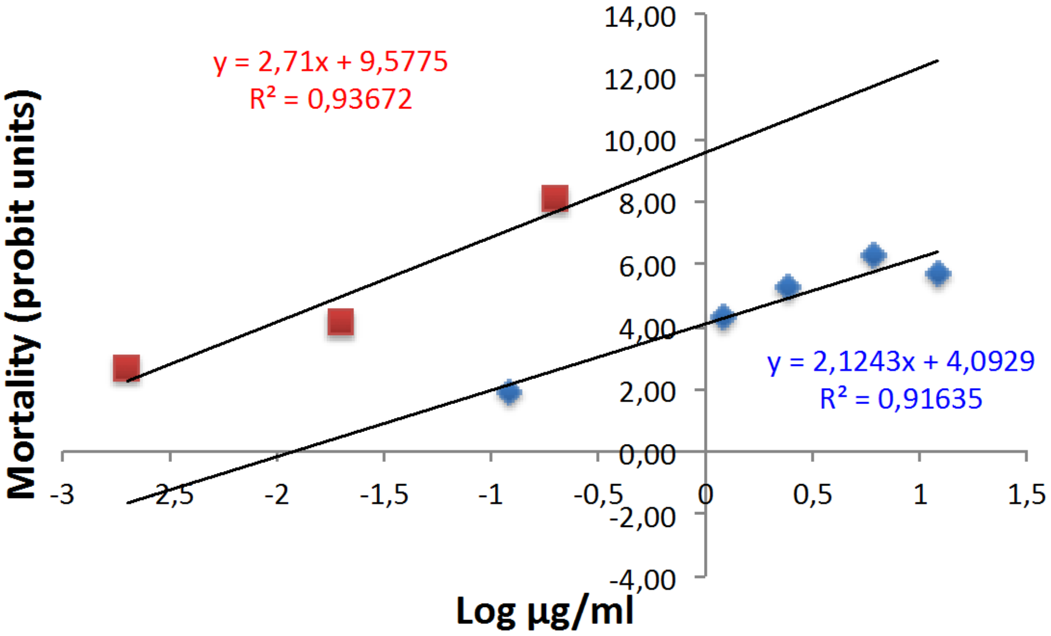

Supplementary Figure 1. LD_{50} determination in *Litopenaeus stylirostris* shrimp and wax moth *Galleria mellonella*. Three (0.01 ng to 0.01 µg protein g^{-1}) and five (12 ng to 1.2 µg protein g^{-1}) concentrations of nigritoxin were tested for *L. stylirostris* (red dots) and *G. mellonella* (blue dots) respectively. Values were calculated using Probit analysis.

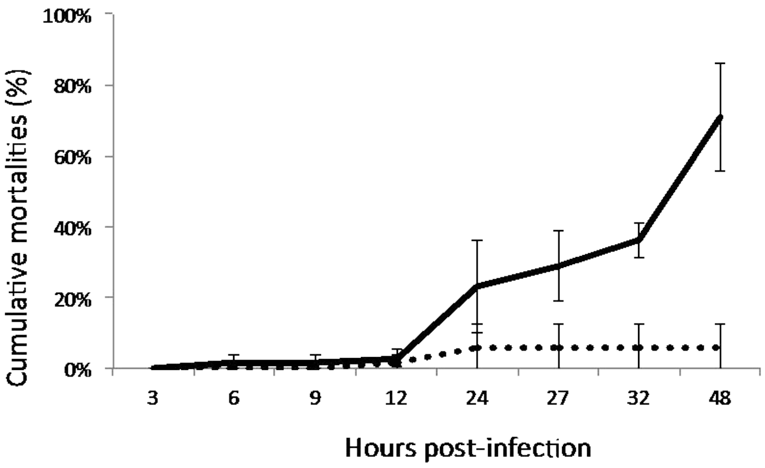

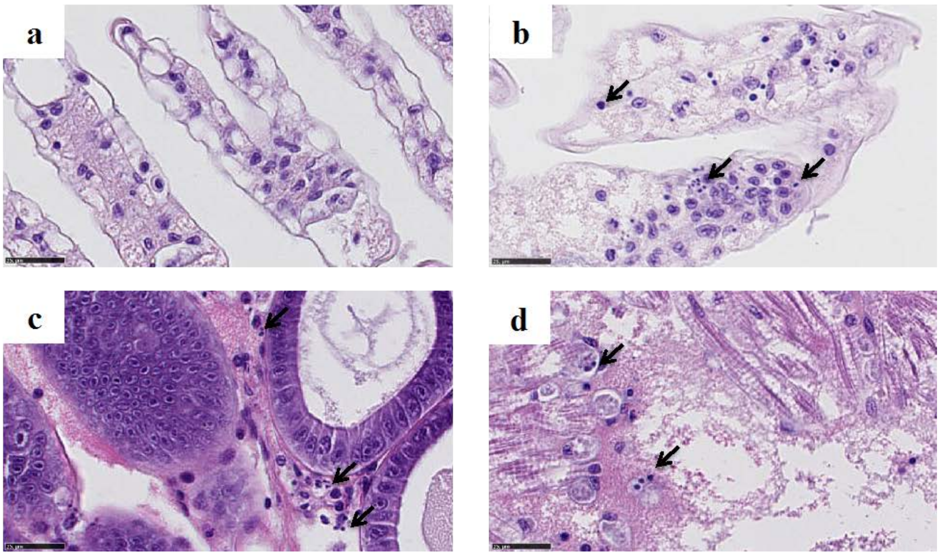

Supplementary Figure 2. Histopathological signs associated to *V. nigripulchritudo* infection. Upper figure: shrimp (*n* = 35, in triplicate) were infected by transient immersion with virulent (strain SFn1, black line) or non-virulent (strain VN110, hatched line) strains of *V. nigripulchritudo*. Mortalities were recorded for 48 hours. **Lower figure:** haematoxylin and eosin stained sections from shrimp sampled 48-h post-infection. Shrimp infected by non-virulent strain exhibited normal circulating hemocytes (a) whereas numerous pyknotic and karyorrhectic nuclei were observed in the hemocytes circulating in the gills (b), within the tubules of the digestive gland (c) and in the heart of infected shrimp (d). Abnormal-shaped nuclei are indicated by black arrows (scale bar: 25 µm).

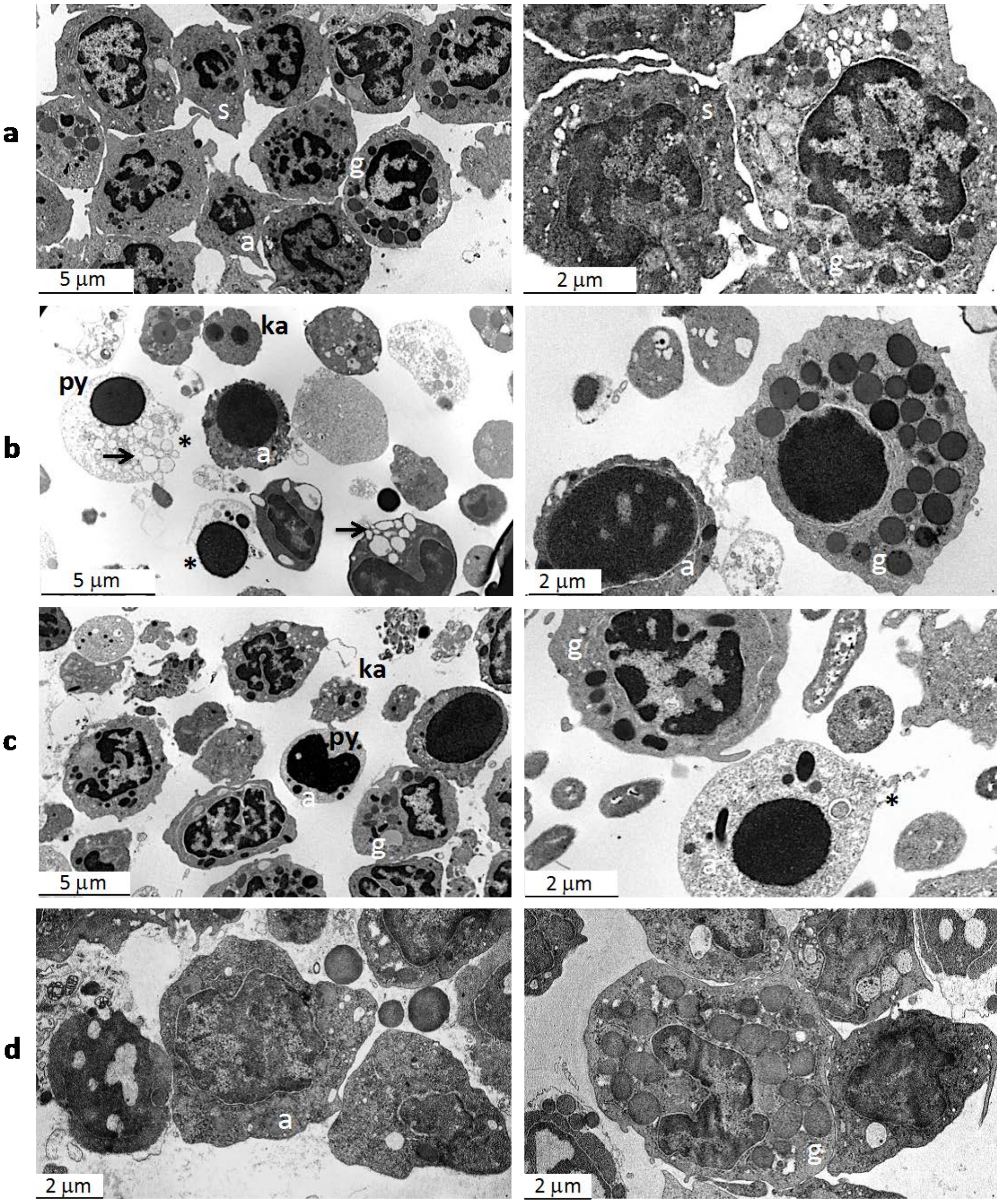

Supplementary Figure 3. Representative TEM images showing morphological and nuclear changes observed in hemocytes (a: agranular or hyaline; s: semigranular; g: granular) collected from *L. stylirostris* shrimp after intramuscular injection of (**a**) saline, (**b**) a *V. nigripulchritudo* virulent strain, (**c**) nigritoxin and (**d**) a non-virulent *V. nigripulchritudo* strain. The hemocytes were collected from shrimp 24 h after injection with *V. nigripulchritudo* or with saline, and 9 h after nigritoxin injection. Abnormal-shaped nuclei showing signs of pyknosis (py) or karyorrhexis (ka) are indicated. Cell lysis and vacuolization are indicated by stars and black arrows, respectively. Scale bars of images are 5 µm or 2 µm as indicated.

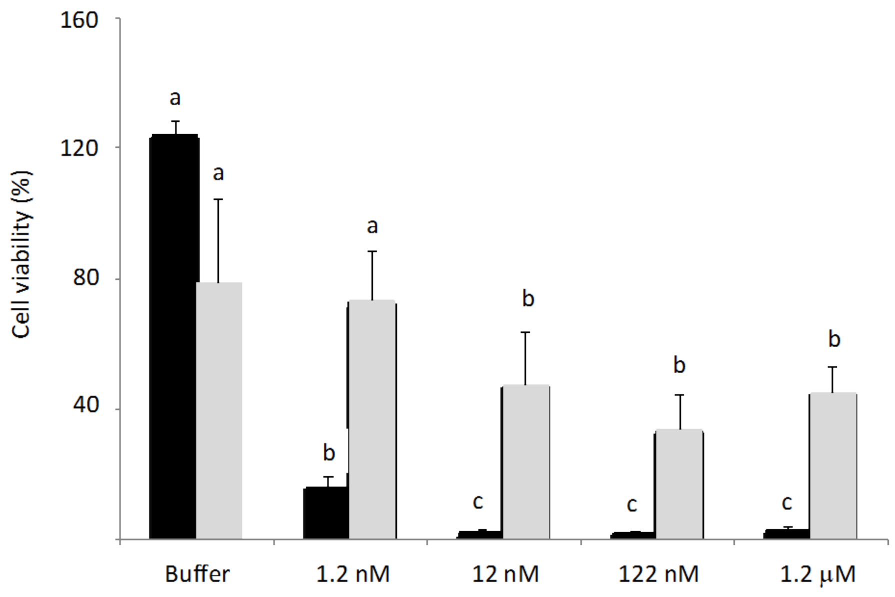

Supplementary Figure 4. Concentration-dependent cytotoxic activity of nigritoxin on insect cell lines. Insect cells (Sf9, black bars and S2, grey bars) were incubated with different concentrations of nigritoxin (indicated on the x axis) or with the same volume of protein suspension buffer for 12 hours. The cytotoxicity was monitored using Alamar Blue (Invitrogen) assay. For each condition, the cell viability was compared to mock cells and expressed in percentage (y axis). In the buffer-treated cells, a percentage of cell viability higher than 100% most likely results from cell division occurring during incubation time. The experiment was performed twice in triplicate and data are presented as mean ± S.D. Means with the same letter are not significantly different from each other (ANOVA, $p<0.05$).

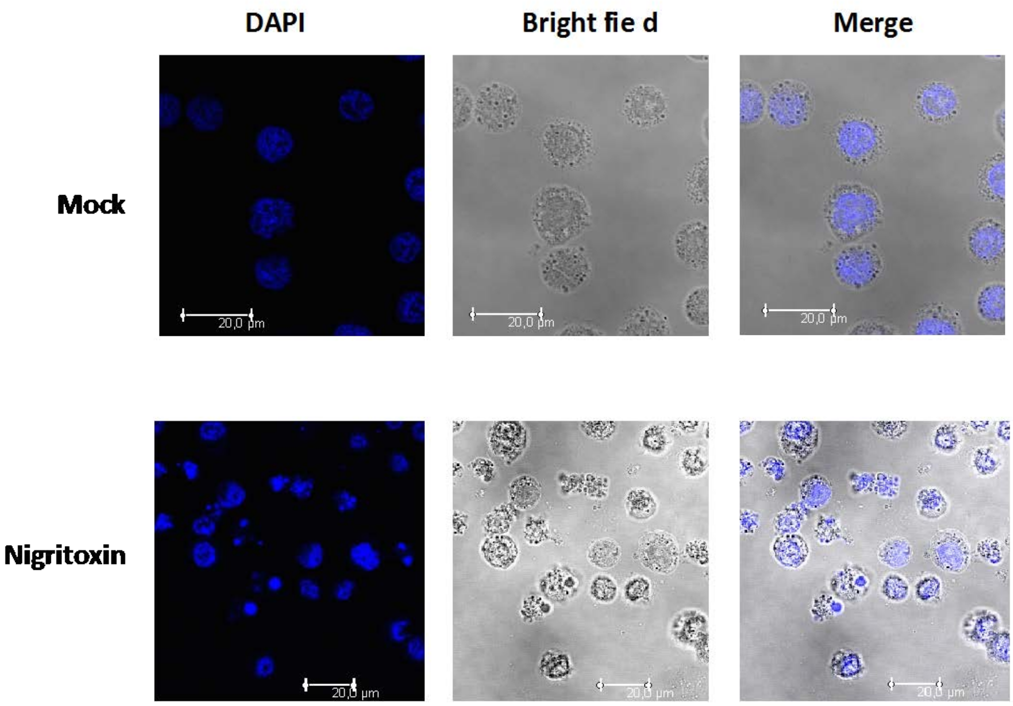

Supplementary Figure 5. Nigritoxin induces nuclei condensation and fragmentation in Sf9 cells. Confocal imaging of Sf9 incubated for 12 h in the absence or in the presence of nigritoxin (1.2 µm, final concentration). DAPI was used to stain cell nuclei. Scale bars, 20 µm.

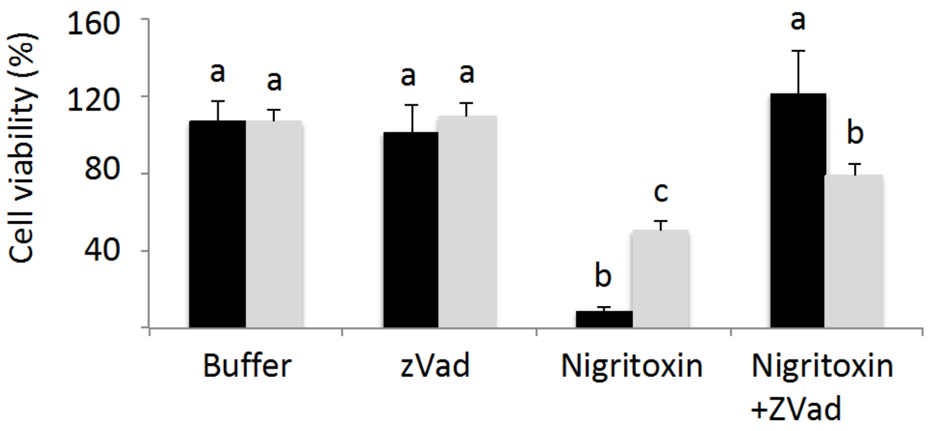

Supplementary Figure 6. Effects of a pan-caspase inhibitor, zVAD-fmk (zVAD) on nigritoxin-induced cell death. Sf9 and S2 cells (black and grey bars, respectively) were pretreated or not with zVAD (50 µM) for 1 h followed by treatment with nigritoxin (1.2 µM) for 12 h. The experiment was performed twice in triplicate and data are presented as mean ± S.D. Means with the same letter are not significantly different from each other (ANOVA, $p<0.05$).

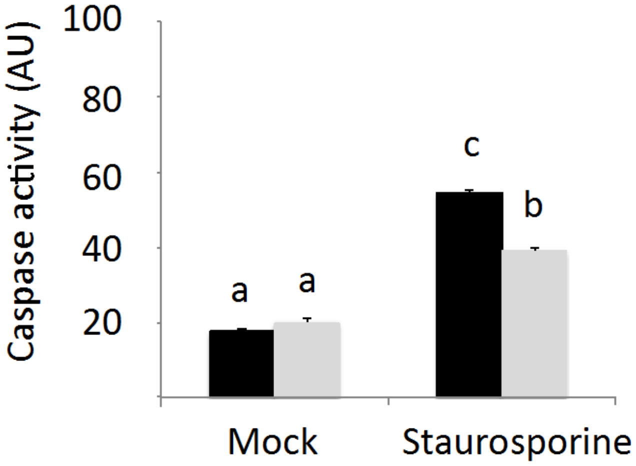

Supplementary Figure 7. Staurosporine-induced caspase activity. Sf9 and S2 cells (black and grey bars, respectively) were either mock-treated or incubated with 1 μM staurosporine for 12 h. Caspase activity was determined using Ac-DEVD-AFC as a substrate. The experiment was performed twice in triplicate and data are presented as mean ± S.D. Means with the same letter are not significantly different from each other (ANOVA, $p<0.05$).

a

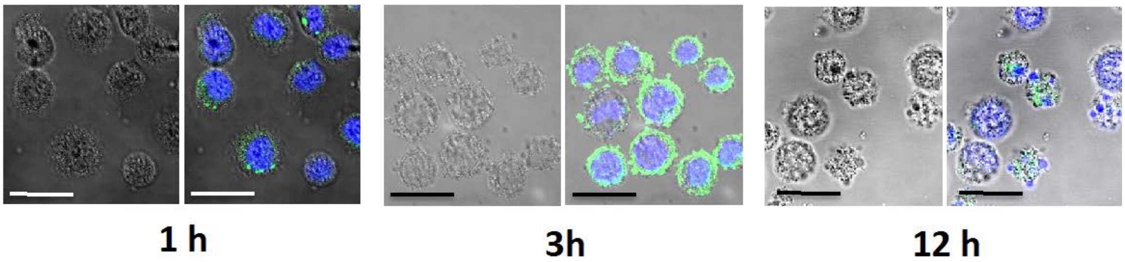

b

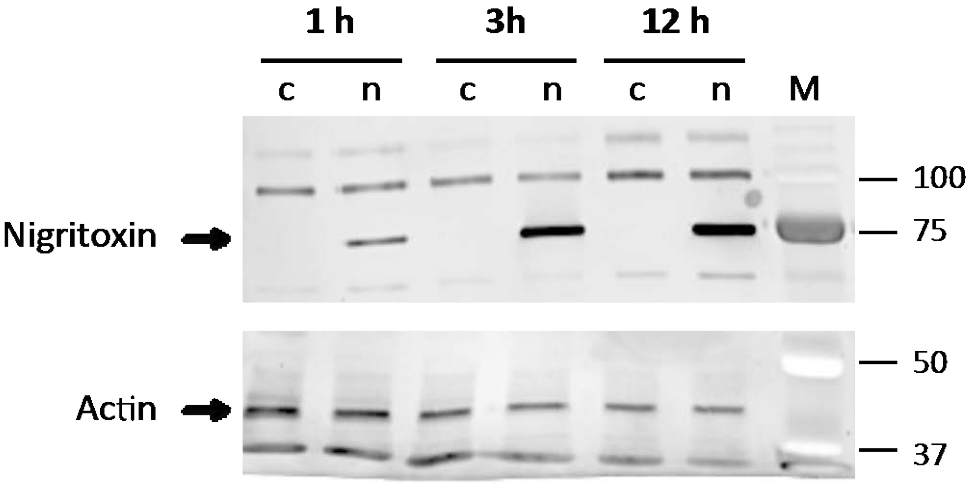

Supplementary Figure 8. Cellular localization of the nigritoxin over time. a) Sf9 cells were incubated with the nigritoxin (1.2 µM) for indicated time, fixed and reacted with a specific anti-nigritoxin polyclonal antibody and Alexa Fluor-488-conjugated secondary antibody (green). Left, bright field image, right, merge image (anti-nigritoxin antibody + DAPI + bright field). Scale bars, 20 µm. **b)** Western-blot analysis of nigritoxin incubated with Sf9 cells. 1.2 µM nigritoxin (n) or an equivalent volume of protein susension buffer (c) was added to Sf9 cells for 1, 3 and 12 h before cell lysis, SDS-PAGE analysis and Western blotting. The molecular masses of marker (M) are indicated on the right (in kDa). An arrow indicates the position of the nigritoxin. β-actin was used as a loading control.

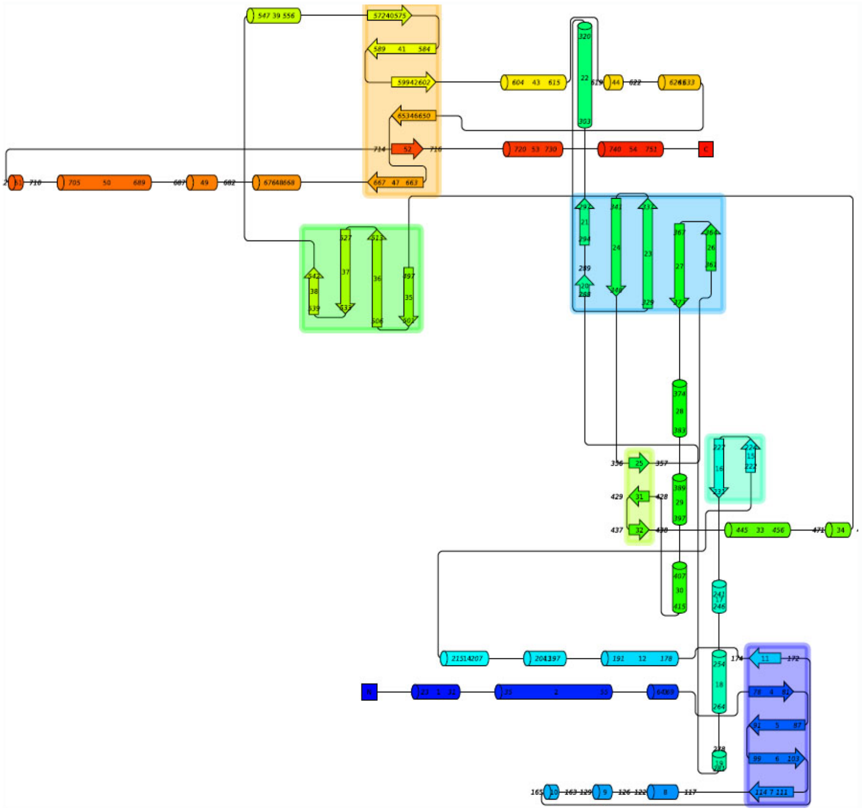

Supplementary Figure 9. The protein fold of nigritoxin contains 28 α-helices and 25 β-strands. All three globular domains of the nigritoxin protein are of mixed α/β type. The different β-strands form four larger anti-parallel β-sheets (containing 4 to 6 strands) and two smaller anti-parallel β-sheets (with 2 to 3 strands) and are distributed among the 3 globular domains as follows: the N-term domain contains one 5-stranded antiparallel β-sheet stacked against 5 large helices and surrounded by 7 other smaller helices. The 5 large helices are at the interface with the central domain that is made up of one large 5-stranded β-sheet and a smaller sheet composed of 3 short strands. This central part of the protein also contains 4 helices. The central domain is connected to the C-term region through 30 residues, 444 to 474, that first form a rather isolated α-helix (residues 444 to 460), followed by a straight, highly disordered stretch (electron density for these residues is barely visible), ending in the first C-term β-sheet composed of 4 strands. A single helix connects this first C-term β-sheet to a second larger one, containing 5 strands that are surrounded by 6 α-helices. The figure was produced using the online program Pro-Origami.

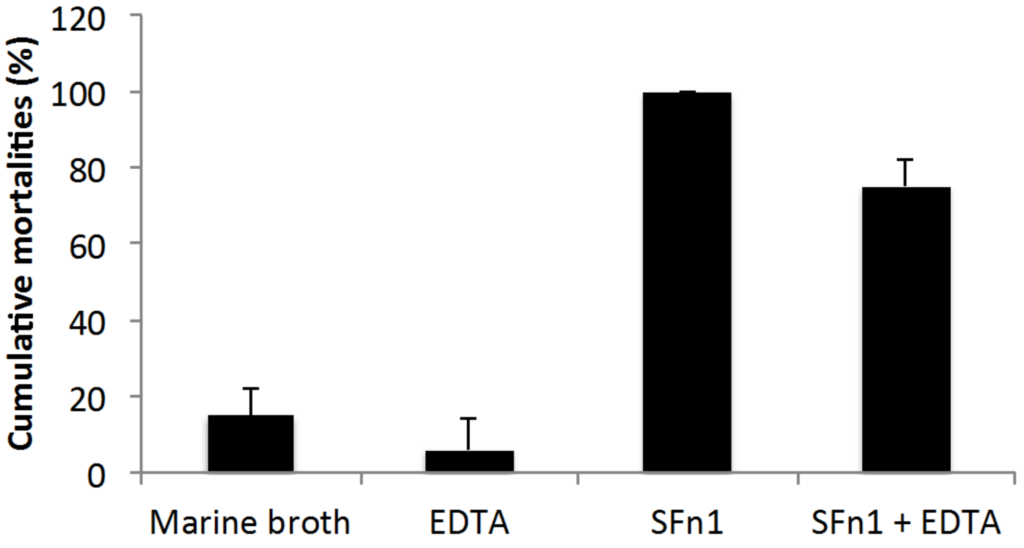

Supplementary Figure 10: Effect of EDTA on *V. nigripulchritudo* culture supernatant toxicity. Bacterial supernatants were prepared from a *V. nigripulchritudo* virulent strain and incubated when indicated for 1 h with the chelating agent EDTA (50 mM, final concentration). *L. stylirostris* shrimp (*n* = 10, in duplicate) were then injected with 100 µl of the supernatant treated of not by EDTA, or as controls, EDTA and marine broth (x axis). Mortalities were recorded for 48 hours.

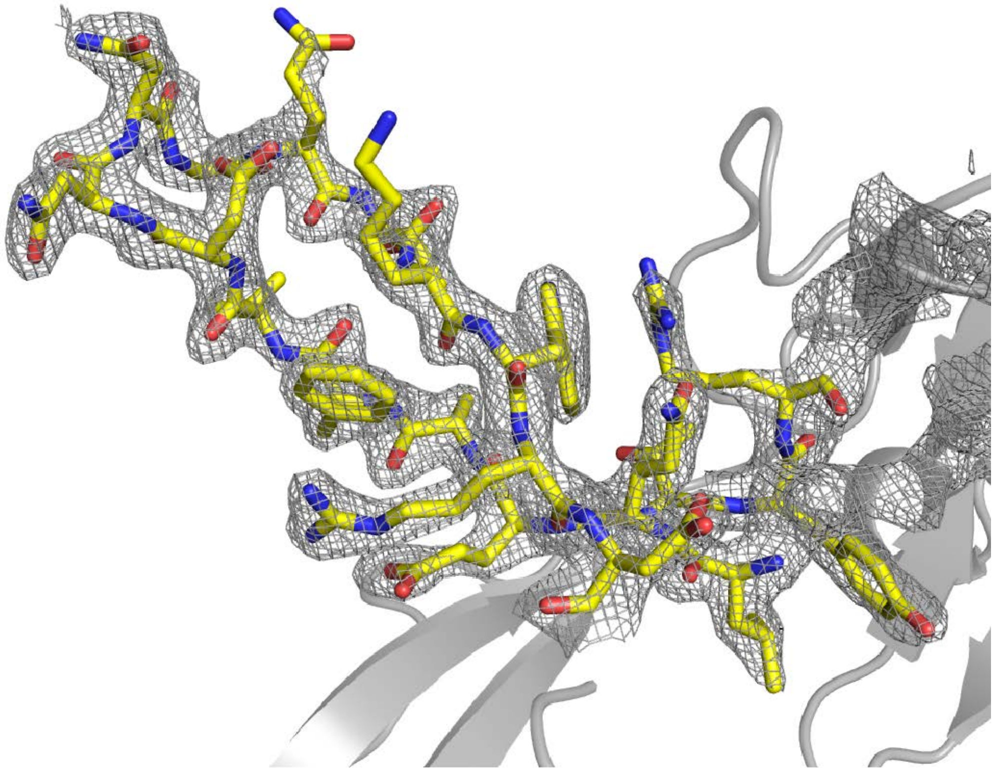

Supplementary Figure 11. Representative electron density map (2Fo-Fc) calculated from the final refined coordinates of residues 510 to 542, showing one of the segments extruding from the molecular surface (‘rabbit ears’). The density map is displayed at a 1.8 σ level.

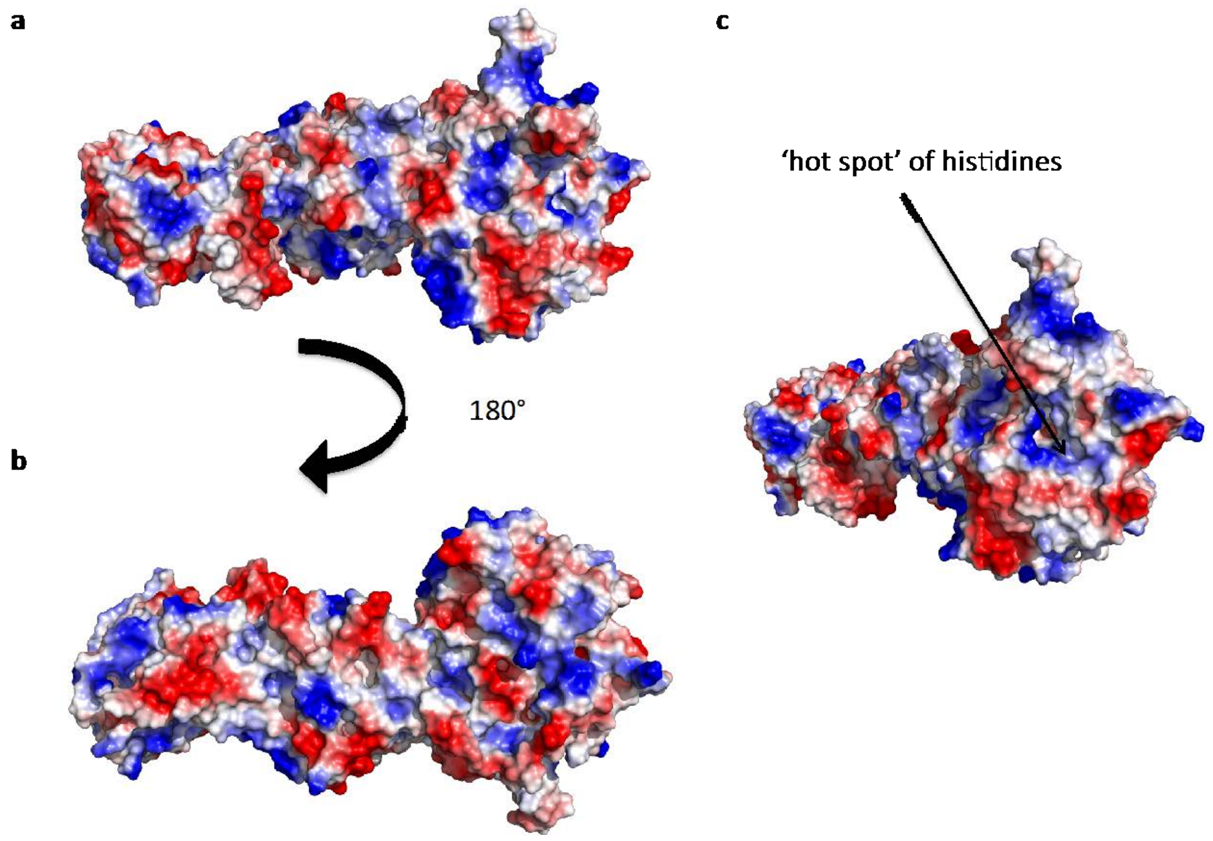

Supplementary Figure 12. Surface representation of the nigritoxin molecule. Three different orientations of the molecule are shown that display the electrostatic surface-potential (a) on the front face, (b) on the back-face turned by 180° with respect to (a), and (c) oriented such that the C-terminal domain is in the first plane. A negatively charged surface-potential is colored in red, positive in blue and the white color represents a neutral surface potential.

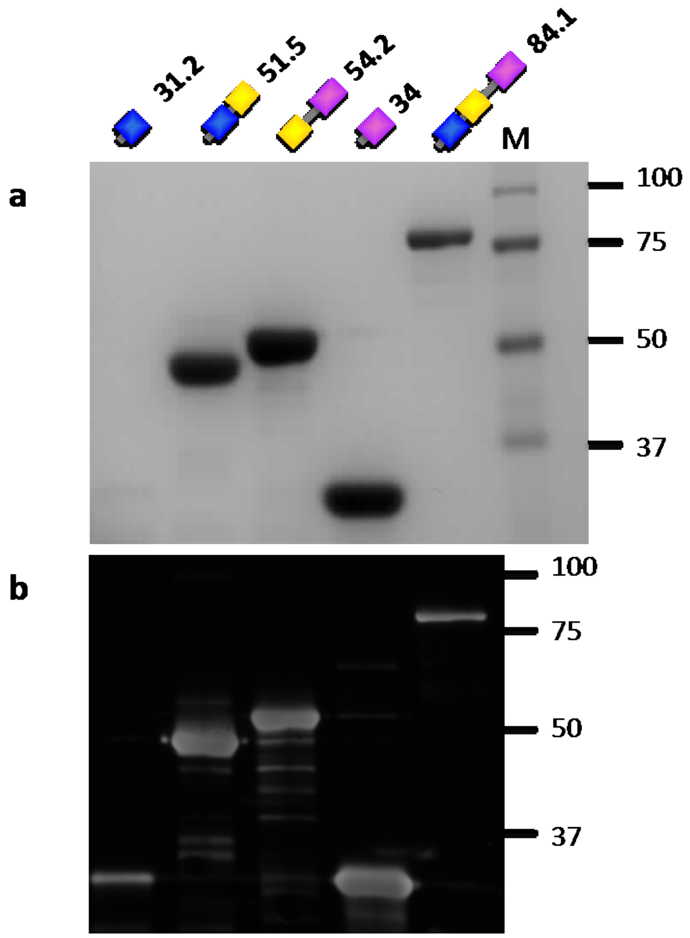

Supplementary Figure 13. SDS PAGE and Western-blot analysis of the His-tagged nigritoxin and truncated versions. The nigritoxin domains are schematically represented in blue: N-terminal; yellow: central; magenta: C-terminal and expected sizes in kDa are mentioned. The His-tagged purified proteins were subjected to SDS/12% PAGE and stained by Coomassie blue (a) or electroblotted onto nitrocellulose (b). The truncated proteins were immunologically detected by incubating the blot with a rabbit anti-nigritoxin polyclonal antibody. The molecular masses of marker (M) are indicated on the left (in kDa).

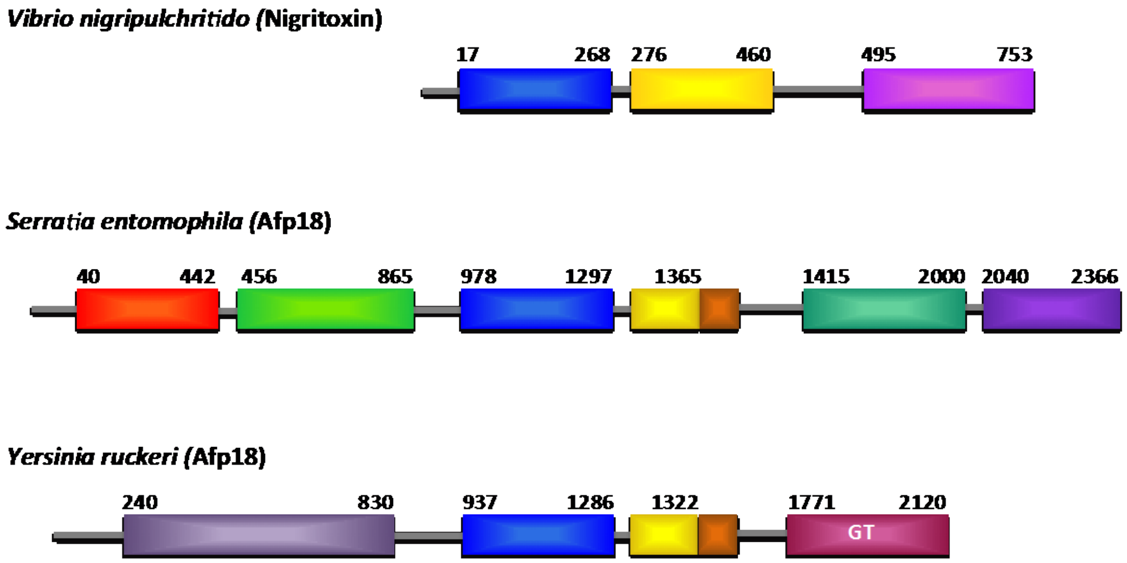

Supplementary Figure 14. Schematic representation of antifeeding prophage 18 (Afp18) from *Serratia entomophila* (accession code WP_010895820.1) and *Yersinia ruckeri* Afp18 (UniProt accession code C4ULG3.1) that contain the conserved one-and-a-half N-term domains, shared with nigritoxin (accession code WP_013610328.1). The domains are colored with a different color when they don't display any sequence similarity. This representation illustrates that the overall domain structure of these proteins is highly variable. Besides nigritoxin, the only other protein domain that has been characterized so far is the glycosyltransferase (GT) domain (1771-2120) from *Y. ruckeri* Afp18.

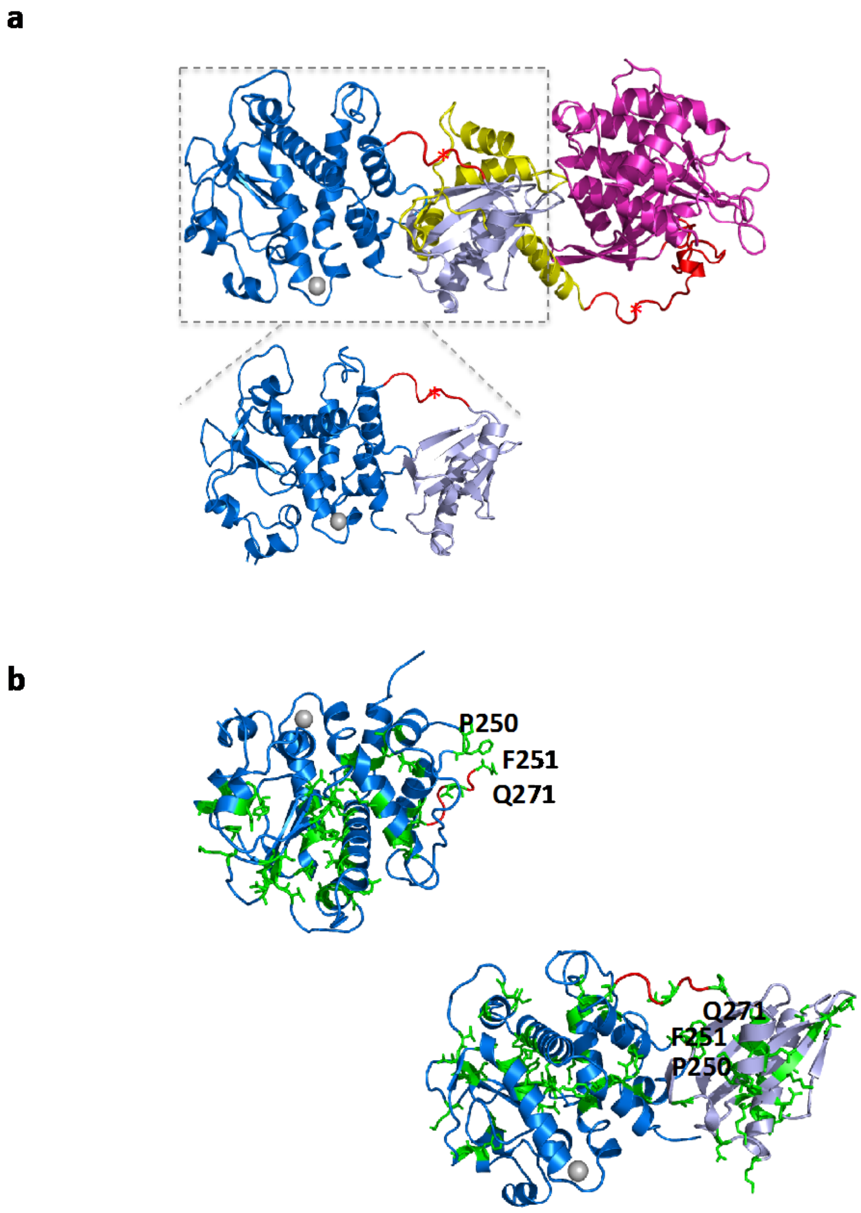

Supplementary Figure 15. Ribbon representation of the N-terminal domain and part of central domain of nigritoxin. This part is conserved in other toxins. **a)** Upper panel, full length nigritoxin: the N-term domain is colored in blue; the part of the central domain that is found in Afp18 and other hypothetical proteins is colored in grey, while the continuation of the central domain of nigritoxin is colored in yellow; the unique C-term domain of nigritoxin is colored in magenta. All linker regions are colored in red. The lower panel shows the extraction of the conserved part with Afp18. This illustrates that the four helices (yellow in upper panel), present in nigritoxin, wrap around the core of the conserved central domain and that the replacement of these in other proteins will lead to quite a different external surface

and consequently, most probably, to a different function. **b)** Upper panel: Ribbon representation of the N-term domain of nigritoxin displaying all strictly conserved residues between protein shown in Fig.S13. Besides P250, F251 and Q271 that are located at the interface of domains 1 and 2, most of the other strictly conserved residues are located in the hydrophobic core of the domain, the side chains are pointing towards the interior of the fold, are rarely exposed to the surface. Lower panel: Ribbon representation of the entire one-and-a-half conserved region, displaying all strictly conserved residues. Within the light blue partial domain again, most of the residue side chains point towards the interior of the fold, although less systematic, since some of these residues are in interaction with structural elements of the second half of the domain (not represented here).

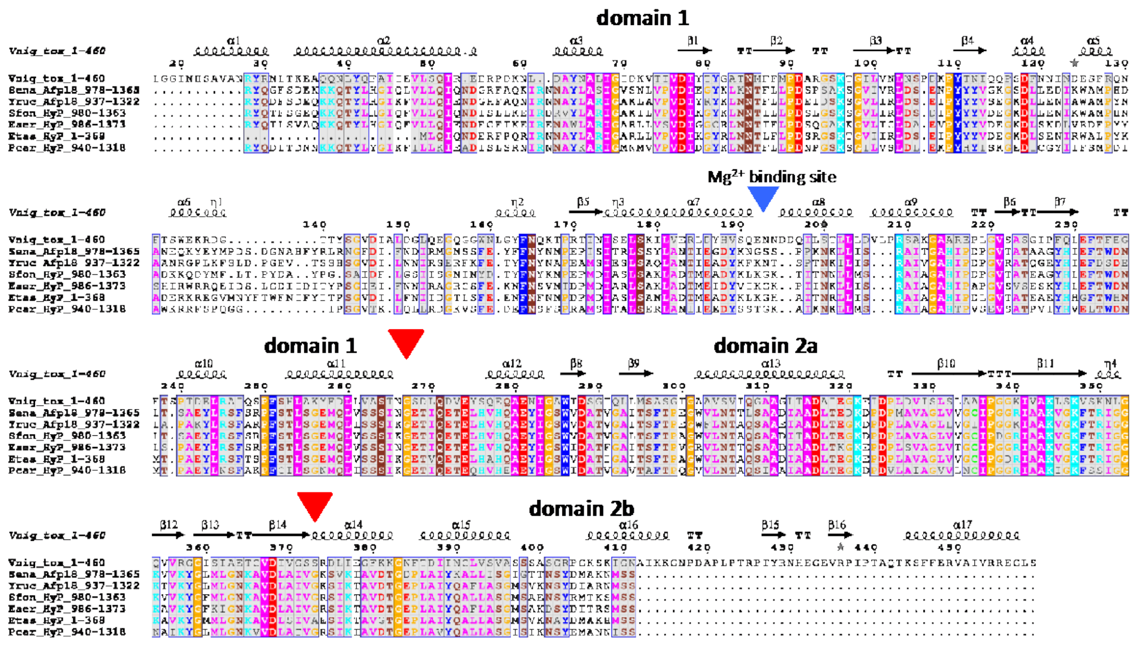

Supplementary Figure 16. Multiple sequence alignment, produced with MULTALIGN and ESPRIPT, showing the extract of the conserved one-and-a-half domains from a selection of proteins and compared to the sequence of nigritoxin (first line). The residue color code corresponds to the nature of the amino acids, colored boxes show the strictly conserved residues, simple boxes highlight residues conserved in most sequences. The secondary structure elements (helices α1 to α17, β-strands β1 to β16, TT are tight turns), as observed in the 3D crystal structure of nigritoxin, of the N-term domain (domain 1) and central domain (domain 2a+2b) are given above the sequence. The numbering above the first sequence corresponds to the residue numbers of nigritoxin. The disordered loop (missing in electron density) and the Mg2+ binding site are highlighted in blue. The first red triangle indicates the boundary of the N-term domain, while the second red triangle delimits the conserved part of domain 2 from the second part that displays no sequence similarity to that of the other proteins. The displayed sequences are: Vnig_tox_1-460: residues 1 to 460 of nigritoxin from *Vibrio nigripulchritudo*; Sena_Afp18_978-1365: residues 978 to 1365 of the protein Afp18 from *Serratia entomophila*; Yruc_Afp18_937-1322: residues 937 to 1322 of the protein Afp18 from *Yersinia ruckeri*; Sfon_Hyp_980-1363: residues 980 to 1363 of a hypothetical protein from *Serratia fonticola*; Eaer_Hyp_986-1373: residues 986 to 1373 of the hypothetical protein from *Enterobacter aerogenes*; Etas_Hyp_1-368: residues 1 to 368 of a

hypothetical protein from *Erwinia tasmaniensis*; Pcar_Hyp_940-1318: residues 940 to 1318 of a hypothetical protein from *Pectobacterium carotovorum*.

Supplementary Tables

Supplementary Table 1: *per-os* inoculation of nigritoxin. The challenge was administered by feeding individually housed shrimp (*n* = 6) with a portion of tissues either injected with nigritoxin or buffer. Survival was assessed after 24 h.

**Experimental condition**	**Cumulative mortality (%)**
Fed with nigritoxin-injected tissues	0
Fed with buffer-injected tissues	0

Supplementary Table 2: Cell cycle analysis of human cell lines treated with 1.2 μM nigritoxin

**Cell line**	**Treatment**	**% of cells in cycle phase[a]**		
		**G1**	**S**	**G2**
HeLa	Control	66.2 ± 0.5	23.2 ± 1.1	10.6 ± 1
	Nigritoxin	65.7 ± 1.6	23.8 ± 1.4	10.5 ± 0.3
Jurkat	Control	45.7 ± 1.2	42.8 ± 0.5	11.4 ± 0.9
	Nigritoxin	46.6 ± 2.8	43.5 ± 2.3	9.9 ± 1.2

[a] The experiment was performed in triplicate and data are presented as mean ± S.D.

Supplementary Table 3: Strains used in the study

Name	Description	Reference
BL21 (DE3)	F^- *ompT hsdS*$_B$(r_B^- m_B^-) *gal dcm* (DE3)	Novagen, USA
B834 (DE3)	F^- *ompT hsdS*$_B$(r_B^- m_B^-) *gal dcm met* (DE3)	Novagen, USA
TOP10	F- *mcrA Δ(mrr-hsdRMS-mcrBC) φ80lacZΔM15 ΔlacX74 recA1 araD139 Δ(ara-leu)7697 galU galK rpsL(Str*^{R}*) endA1 nupG*	Invitrogen, USA
GV771	BL21 (DE3) + pFO4- nigritoxin (aa 1-757)	This study
GV1295	BL21 (DE3) + pFO4- nigritoxin (aa 1-270)	This study
GV1296	BL21 (DE3) + pFO4- nigritoxin (aa 1-457)	This study
GV1298	BL21 (DE3) + pFO4- nigritoxin (aa 271-757)	This study
GV1299	BL21 (DE3) + pFO4- nigritoxin (aa 486-757)	This study
GV1807	BL21 (DE3) + pFO4-nigritoxin- *Δrabbit ears*	This study
GV1805	BL21 (DE3) + pFO4-nigritoxin H598->A and H650->A	This study
GV1349	BL21 (DE3) + pFO4-nigritoxin R512->A and W540->A	This study

Supplementary Table 4: Primers used in the study

**Name**	**Sequence 5'-3'** [a]
090413-2	CGC**ggatcc**TCTCTACCCTCAAACCC
090413-3	CCG**ctcgag**TTAAACTGATGATGAAGCC
290914-1	CGC**ggatcc**TTAACTGCAGCACAAAAAAG
290914-2	CCG**ctcgag**TTAGAGATCCGAACCATTAG
290914-3	CCG**ctcgag**TTAGTCAAGACCGATAGCC
200315-1	**ggatcc**ATGGTGATGGTGATG
200315-2	**ctcgag**GCTAGCATGCATG
200315-3	CATCACCATCACCAT**ggatcc**CAAGATGTGGAATACAGCC
200315-5	CATGCATGCTAGC**ctcgag**TTAAACTGATGATGAAGC
060515-2	GAAAAAAACATTACTTAT**GCG**AAGGTCCCAGTTCCAAACG
060515-3	GTTTGGAACTGGGACCTT**CGC**ATAAGTAATGTTTTTTTC
060515-4	AATGGTCAAACCAAA**GCG**CGTGATTTTCCCTACG
060515-5	CGTAGGGAAAATCACG**CGC**TTTGGTTTGACCATTG
310816-3	GGGATAAGTAATAAC**GCG**TTAACGTTGGAGAG
310816-4	CTCTCCAACGTTAA**CGC**GTTATTACTTATCCC
310816-5	GATATTCCTGACAACATT**GCG**TTTCGGACTGACCGAGAG
310816-6	CTCTCGGTCAGTCCGAAA**CGC**AATGTTGTCAGGAATATC
310816-11	GTAGGGAAAATC**GCCGCCGCC**AATGTTTTTTTCAATACCTT CTGCTTC
310816-12	GAAAAAAACATT**GGCGGCGGC**GATTTTCCCTACGCTGGTGA AGAGTTTG

[a] Base changes are indicated in red; restriction site are indicated in bold.
